# Lactate infusion as therapeutical intervention: a scoping review

**DOI:** 10.1007/s00431-022-04446-3

**Published:** 2022-03-18

**Authors:** Loes A. van Gemert, Bastiaan E. de Galan, Ron A. Wevers, Rob ter Heine, Michèl A. Willemsen

**Affiliations:** 1 Department of Pediatric Neurology, Amalia Children’s Hospital, Radboud University Medical Center, Nijmegen, the Netherlands; 2 Department of Internal Medicine, Radboud University Medical Center, Nijmegen, the Netherlands; 3 Department of Internal Medicine, Maastricht UMC+, Maastricht, the Netherlands; 4 CARIM School for Cardiovascular Diseases, Maastricht University, Maastricht, the Netherlands; 5 Department of Laboratory Medicine, Radboud University Medical Center, Nijmegen, the Netherlands; 6 Department of Pharmacy, Radboud University Medical Center, Nijmegen, the Netherlands; 7 Donders Institute for Brain, Cognition and Behaviour, Nijmegen, the Netherlands

**Keywords:** Sodium lactate, Brain energy metabolism, Therapeutic lactate infusion, Review

## Abstract

Traditionally, clinicians consider lactate as a waste product of anaerobic glycolysis. Interestingly, research has shown that lactate may serve as an alternative fuel for the brain to protect it against harm. The increasing scientific awareness of the potential beneficial side of lactate, however, is entering the clinic rather slowly. Following this, and realizing that the application of potential novel therapeutic strategies in pediatric populations often lags behind the development in adults, this review summarizes the key data on therapeutic use of intravenous infusion of sodium lactate in humans. PubMed and clinicaltrial.gov were searched up until November 2021 focusing on interventional studies in humans. Thirty-four articles were included in this review, with protocols of lactate infusion in adults with diabetes mellitus, traumatic brain injury, Alzheimer’s disease, and cardiac disease. One study on lactate infusion in children was also included. Results of our literature search show that sodium lactate can be safely administrated, without major side effects. Additionally, the present literature clearly shows the potential benefits of therapeutic lactate infusion under certain pathological circumstances, including rather common clinical conditions like traumatic brain injury.

*Conclusion*: This review shows that lactate is a save, alternative energy source for the adult brain warranting studies on the potential therapeutic effects of sodium lactate infusion in children.

**Table:** 

**What is Known:** *• Lactate is generally considered a waste product of anaerobic glycolysis. However, lactate also is an alternative fuel for different organs, including the brain.* *• Lactate infusion is not incorporated in standard care for any patient population.*	
**What is New:** *• Thirty-four studies investigated the therapeutic use of intravenous sodium lactate in different patient populations, all with different study protocols.* *• Literature shows that lactate infusion may have beneficial effects in case of hypoglycemia, traumatic brain injury, and cardiac failure without the risk of major side effects.*

**Supplementary information:**

The online version contains supplementary material available at 10.1007/s00431-022-04446-3.

## Introduction

In some rare inborn errors of metabolism (e.g., glucose transporter 1 deficiency syndrome (Glut1DS) and glycogen storage disease 1 (GSD1)) and under certain clinical circumstances (e.g., recurrent hypoglycemia), lactate may serve as a fuel to the brain [1]. This knowledge could be of use in developing treatment options for various neurometabolic disorders. However, a persisting negative connotation of lactate can be found almost everywhere else in medicine, especially in clinical practice since an increasing level of lactate in blood is a well-known marker for severe illness and hypoxia. The negative connotation of lactate can also be explained in a historical context. Lactate was first discovered in 1780 by Scheele [2] and in 1808, Berzelius found an elevated concentration of lactate in “the muscles of hunted stags.” A century and many studies on lactate later [3], Fletcher and Hopkins reported in their landmark paper in 1907 that the level of lactate in muscles is low in resting conditions, increases under conditions of hypoxia or when muscles are activated, and decreases again when placed in an oxygen-rich environment [4]. The theory that lactate production and accumulation are a result of the insufficient availability of oxygen became generally accepted.

Nonetheless, more recent studies again showed the other, beneficial side of lactate. In the eighties of last century, Brooks et al. formulated the “cell to cell lactate shuttle hypothesis,” which is based on the principle that one cell may excrete lactate that is formed after glycolysis, while another cell may take up and further process lactate for the benefit of energy metabolism [5, 6]. Other studies have also shown the beneficial side of lactate as an alternative energy source for different organs, including the brain [7, 8]. Lactate can cross the blood–brain barrier, and when oxidized in the brain, it can provide up to 8–10% of the brain’s energy requirement. This contribution could even be larger when the concentration of lactate in blood is artificially raised by lactate infusion [9–11]. Pellerin and Magistretti developed the astrocyte neuron lactate shuttle (ANLS) theory, which proposes that astrocytes take up glucose, turn it into lactate through aerobic glycolysis, and transport it to neurons, where it can be used as a source of energy [12, 13]. Thereafter, many studies concluded that their results are in accordance with the ANLS theory [14–17].

Unfortunately, there is still little attention for lactate and its beneficial side in clinical practice. The aim of this review is to thoroughly review available literature on the use of lactate in a clinical setting and to provide an overview of protocols infusing lactate in different patient groups. By doing so, we intend to bring attention to the potential therapeutic effects of lactate and to catalyze the discussion on the possible benefits of therapeutic lactate infusion among pediatricians and other pediatric specialists. We hope to increase the awareness of the therapeutic use of lactate and to facilitate the development of future clinical trial protocols in pediatric populations, which so often tremendously lag behind the developments in adults.

## Materials and methods

A scoping review was performed, focusing on study designs and used protocols of lactate infusion in humans. The PRISMA guidelines were followed wherever possible (see supplemental text for the checklist and exact MeSH terms) [18]. We searched PubMed up to November 2021, and searched on clinicaltrial.gov for additional relevant studies not yet published. Limits were used for “English” and “Dutch.” After correction for duplicates, we identified 282 articles. Following screening of title and abstracts of all articles, 42 relevant articles remained. Once we checked references of all articles after 2010, we found another 76 articles, 9 of which were included. Exclusion criteria were articles without the aim to increase normal lactate concentration in blood and articles that did not infuse lactate in humans in a clinical setting. After applying these exclusion criteria, 34 articles remained; only one of which involved children. We abstracted data on patient characteristics (diagnosis, number of patients included), protocol details (infusion concentration, rate and duration, as well as total infused lactate, and peak blood concentration of lactate), and reported beneficial and adverse effects.

## Results

The included studies are listed in Table 1. In all studies, solutions of sodium lactate were intravenously administered, except in the studies of Gallagher et al. and Jalloh et al. who used a technique based on cerebral microdialysis. Unless otherwise indicated, all studies included adults in their research.

**Table 1:** Study designs and observed biochemical side effects of sodium lactate infusion

	***Author***	***Conc. infusion (mmol/L)***	***Rate infusion (mmol/kg/min)***	***Duration infusion (min)***	***Peak blood conc. lactate (mmol/L)***	***Total infused lactate (mmol/kg)***	***Total infused sodium (mmol/kg)***	***pH increase***	***Sodium increase (mmol/L)***	***Bicarbonate increase (mmol/L)***	***Potassium decrease (mmol/L)***	***Chloride decrease (mmol/L)***	***Phosphate decrease (mmol/L)***
DM	Maran et al. [19]	0.6	0.03	140	2.4	4.2	4.2	0.10	^b^	^b^	^b^	^b^	^b^
	Veneman et al. [20]	^b^	0.05 + 0.03	20 + 70	5.4	3.1	3.1	^b^	^b^	^b^	^b^	^b^	^b^
	King et al. [21]	600	0.04	125	3.3	5	5	^b^	^b^	^b^	^b^	^b^	^b^
	King et al. [22]	600	0.04	125	3.5	5	5	^b^	^b^	^b^	^b^	^b^	^b^
	Maran et al. [23]	^b^	0.03	60	1.8	1.8	1.8	^b^	^b^	^b^	^b^	^b^	^b^
	De Feyter et al. [24]	^b^	0.01	105	1.8	1.05	^d^	^b^	^b^	^b^	^b^	^b^	^b^
	Wiegers et al. [25]	600	A) 0.05 + 0.03 B) 0.04 + 0.025	A) 15 + 85 B) 15 + 85	3.5	A) 3.3 B) 2.725	A) 3.3 B) 2.725	0.07	^b^	^b^	^b^	^b^	^b^
TBI	Gallagher et al. [11]^a^	4	1.2 × 10^–6 c^	^b^	^b^	^d^	^d^	^b^	^b^	^b^	^b^	^b^	^b^
	Ichai et al. [26]	504.1	0.76	15	2.7	11.3	11.3	100.5%^f^	101.4%^f^	^b^	^b^	^b^	^b^
	Ichai et al. [27]	500	0.00417	2880	1.5	12	12	0.12	7	^b^	^b^	^b^	^b^
	Bouzat et al. [28]	1000	0.04 + 0.03	60 + 120	5	7.8	7.8	0.04	1.96	^b^	^b^	^b^	^b^
	Glenn et al. [29]	^b^	5.7^c^	90	7.1	513^e^	^d^	^b^	^b^	^b^	^b^	^b^	^b^
	Bisri et al. [30]	504.1	0.013	15	^b^	0.189	0.189	^b^	^b^	^b^	^b^	^b^	^b^
	Quintard et al. [31]	1000	0.04	180	4–5	7.2	7.2	^b^	^b^	^b^	^b^	^b^	^b^
	Carteron et al. [32]	1000	0.03	180	5.1	5.4	5.4	^b^	6	^b^	^b^	^b^	^b^
	Jalloh et al. [33]^a^	8	2.4 × 10^–6 c^	^b^	^b^	^d^	^d^	^b^	3	^b^	^b^	^b^	^b^
	Wolahan et al. [34]	890	0.5 + 0.163	5 + 180	1.9	29.34	29.34	^b^	^b^	^b^	^b^	^b^	^b^
AD	Járdánházy et al. [35]	500	0.125	20	^b^	2.5	2.5	^b^	^b^	^b^	^b^	^b^	^b^
	Kálmán et al. [36]	500	0.125	20	^b^	2.5	2.5	^b^	^b^	^b^	^b^	^b^	^b^
	Kálmán et al. [37]	500	0.125	20	5.5	2.5	2.5	^b^	^b^	^b^	^b^	^b^	^b^
CD	Boom et al. [40]	504.1	0.1	15	6.1	0.15	0.15	0.08	2	1.2	^b^	^b^	^b^
	Leverve et al. [41]	504.1	0.013	720	4.6	9.6	9.6	^b^	2	b	^b^	^b^	^b^
	Mustafa and Leverve [42]	1000	0.17	15	14.8	2.5	2.5	0.04	5	1.9	^b^	^b^	^b^
	Nalos et al. [43]	500	0.1 + 0.0083	15 + 1440	4.5	12.15	12.15	0.05	10	2.6	0.9	6	0.25
DS	Somasetia et al. [44]	500	0.167 + 0.008	15 + 720	2.9	8.3	8.3	0.1^g^	0.8	11.3^g^	1.2	4.8	^b^
PA	Dager et al. [49]	500	0.25	20	10	5	5	^b^	^b^	^b^	^b^	^b^	^b^
	Dager et al. [48]	500	0.25	20	^b^	5	5	^b^	^b^	^b^	^b^	^b^	^b^
	Dager et al. [50]	500	0.25	20	10.8	5	5	^b^	^b^	^b^	^b^	^b^	^b^
	Dager et al. [51]	500	0.25	20	^b^	5	5	^b^	^b^	^b^	^b^	^b^	^b^
	Friedman et al. [52]	500	0.25	20	^b^	5	5	^b^	^b^	^b^	^b^	^b^	^b^
	Layton et al. [53]	500	0.25	20	^b^	5	5	^b^	^b^	^b^	^b^	^b^	^b^
	Otte et al. [54]	500	0.25	20	^b^	5	5	^b^	^b^	^b^	^b^	^b^	^b^
	van der Molen et al. [55]	500	0.25	20	^b^	5	5	^b^	^b^	^b^	^b^	^b^	^b^
	Keck et al. [56]	500	0.25	20	^b^	5	5	^b^	^b^	^b^	^b^	^b^	^b^

*DM* diabetes mellitus, *TBI* traumatic brain injury, *AD* Alzheimer’s disease, *CD* cardiac disease, *DS* dengue shock, *PA* panic attack

^a^Gallagher et al. and Jalloh et al. infused lactate via cerebral microdialysis, not intravenously

^b^No information in article

^c^Infusion mmol/minute

^d^Valuables cannot be calculated with available information

^e^Total infused lactate mmol

^f^Side effects in article available in percentage of baseline value

^g^Venous measurement

### Lactate as alternative energy source for the brain (*n* = 20/34 articles)

Sodium lactate has been infused to patients with diabetes mellitus [19–25], traumatic brain injury (TBI) [11, 26–34], and Alzheimer’s disease [35–37].

Studies among people with or without diabetes mellitus suggest that the brain is able to use lactate during hypoglycaemia [19–25]. Several studies showed that lactate administration suppresses the counterregulatory hormone and symptom responses to hypoglycemia [19, 20, 23, 25], whereas a fall in brain lactate concentration during hypoglycemia among people with type 1 diabetes and impaired awareness of hypoglycemia also suggests lactate consumption [38]. These studies conclude that lactate infusion reduces the awareness of hypoglycemia and prevents clinical signs and symptoms of neuroglycopenia.

TBI can be divided into primary and secondary injury. Primary injury is defined as brain damage directly resulting from external forces of trauma. Secondary injury develops minutes to days after the acute insult as a result of a complex cascade of metabolic and chemical changes induced by the primary tissue damage [39]. In TBI, a high lactate concentration in plasma predicts poor outcome. Interestingly, Gallagher et al. and Jalloh et al. showed that the brains of patients with and without TBI are able to utilize lactate as energy source [11, 33] when lactate was administered through local microdialysis. Additionally, Glenn et al. reported that the cerebral uptake of glucose in patients with TBI was significantly decreased compared to the control group. Nonetheless, the cerebral uptake of lactate did not differ between both groups and in both groups lactate was oxidized to CO_2_ [29]. Other studies showed that administering lactate was associated with a better long-term outcome than standard care in patients with TBI [26, 30–32, 34]. Moreover, studies showed that infusion of lactate in patients with TBI causes a less severe increase in intracranial pressure, a known and feared complication in TBI [27, 28, 33].

Lactate may lead to vasodilatation in cerebral vasculature [32]. Impaired cerebral microcirculation and glucose metabolism are established markers of Alzheimer’s disease. To our knowledge, three studies have investigated whether lactate could serve as an alternative energy source and positively influence cerebral blood flow in patients with Alzheimer’s disease [35–37]. Unfortunately, it did not seem to improve cognitive functioning of the patients.

### Lactate infusion in cardiology (*n* = 4/34 articles)

It is well known that free fatty acids rather than glucose are the preferred energy substrate for the myocardium, but a stressed myocardium increases the net uptake and utilization of lactate [7]. Four studies have shown that hypertonic lactate infusion induces an increase in cardiac output compared with traditional infusion fluids after cardiac surgery [40–43]. Additionally, this was accomplished with less totally infused volume of sodium lactate than that of the comparator fluid, thus reducing the risk fluid overloads in patients with potentially compromised cardiac function [40–43]. None of the studies measured the uptake of lactate by the myocardium directly.

### Lactate infusion in children (*n* = 1/34 articles)

One study investigated the infusion of hyperosmolar lactate in children with dengue shock syndrome [44]. For fluid resuscitation in dengue shock syndrome, the World Health Organization guideline requires large volumes of Ringer lactate, which might induce fluid overload. The effectiveness of the recommended volume of Ringer lactate versus a smaller volume of hyperosmolar lactate solution were compared and a similar hemodynamic shock recovery and plasma expansion were achieved in both groups, with a significantly lower fluid intake and fluid accumulation in the hyperosmolar lactate solution group, without major side effects. Unfortunately, the authors did not investigate whether lactate was used as an alternative energy source.

### Possible adverse effects of sodium lactate infusion (*n* = 9/34 articles)

In 1967, Pitts and McClure noticed so-called exercise-induced attacks of anxiety in patients with “anxiety neurosis.” They found that during standard workouts, the level of lactate in blood significantly increased, and they postulated that the high concentration of lactate in blood provoked panic attacks [45]. This induced a curiosity for the neurobiological genesis of panic [46]. Up until now, the panic-inducing effect of lactate infusion has been demonstrated in animals and patients with panic attacks. Besides lactate, researchers developed a variety of other means to evoke panic attacks under controlled circumstances [47]. Studies infusing lactate to induce panic attacks generally used the same protocol as the original study of Pitts and McClure, resulting in blood lactate concentrations up to approximately 10 mmol/L [48–56]. However, panic attacks only occurred in studies set up to induce these attacks. No other studies with lactate concentrations up to 15 mmol/L reported panic attacks as an adverse event [42, 57].

An overview of noted adverse effects is shown in Table 1. These adverse effects include changes in the equilibrium of electrolytes, and change in pH using sodium lactate, especially an increase of serum sodium concentration might be a concern. However, only a few studies reported sodium increases in their patients or study participants (see Table 1). We found that all adverse effects, including increase of serum sodium concentrations were minor, none of which requiring treatment or interruption of the lactate infusion [19, 25–28, 32, 33, 40–44].

### Summary of infusion protocols

Reviewing the included studies, we closely paid attention to the used study protocols. A summary of used doses, time of administration, and concentration of lactate in blood can be found in Table 1.

When evaluating the infusion protocols, we excluded the studies of Gallagher et al. and of Jalloh et al. since they did not infuse lactate intravenously [11, 33]. First, studies used an infusion fluid with a concentration of lactate between 400 and 1000 mmol/L (for comparison: Ringer’s lactate has a lactate concentration of 28 mmol/L). The lactate solutions used in the studies were either prepared by the pharmacy department of the investigating hospital, or purchased from an external company in the requested concentration. Second, the infusion rate and time differed greatly between protocols. The median infusion rate was 0.05 mmol of lactate per kg bodyweight per minute (mmol/kg/min) (mean 0.13 mmol/kg/min), with the lowest rate at 0.00417 mmol/kg/min and the highest rate at 0.76 mmol/kg/min. The median infusion time was 60 min (mean 235 min), with the shortest infusion time at 15 min and the longest infusion time 2880 min (48 h). In addition, there was a large variability in infusion rate and time in different studies investigating similar patient populations. Finally, due to the different infusion rate and time, the total amounts of infused lactate and the peak concentration of lactate in blood also varied among studies. The median total amount of lactate infused was 5.0 mmol/kg (mean 5.8 mmol/kg), with the lowest total amount of lactate of 0.15 mmol/kg and the highest total amount of lactate of 29.34 mmol/kg. The mean peak concentration of lactate in blood was 4.9 mmol/L (median 4.5 mmol/L), with the lowest peak concentration at 1.5 mmol/L and the highest at 14.8 mmol/L. None of the protocols explained the chosen lactate concentration, infusion rate or duration. The most reported side effects were changes in the equilibrium of electrolytes. None of the studies reported panic attacks. All side effects were minor, none of the patients needed treatment and the protocol of lactate infusion could continue in all cases.

### Protocol for future studies

The protocols of the included studies (see Table 1) can help when drafting a protocol for a future study, but it is impossible to provide a protocol that would fit all future trials in pediatric patients. Besides the fact that children are not little adults, the details of such a protocol, e.g., the duration of infusion and the monitoring of specific parameters, would obviously depend on the disorder involved, and the aim of the study.

Previous studies have shown that the concentration of lactate in the brain increased rapidly following the raise of its blood concentration [9, 10]. For that reason it is suggested, when conducting a study to investigate lactate as an alternative energy source for the brain, to start the infusion with a bolus of sodium lactate for priming, followed by a continuous (but lower) infusion rate for the remainder of the study. In children, when infusing lactate for a limited duration of time, we consider it safe to target concentrations of lactate in blood that are normally reached during strenuous but physiological exercise, i.e., between 7.5 and 10 mmol/L [58].

When infusing lactate in children in a future study, we would advise to monitor vital signs including heartrate, respiratory rate, and blood pressure and specific parameters depending on the condition that is investigated. In relation to the possible adverse effect, we suggest to take frequent blood samples at baseline, during the infusion time and after infusion of lactate, to monitor pH and base excess, as well as concentrations of sodium, potassium, chloride, bicarbonate, lactate, and glucose in blood. Because of the sodium load, we would not include children with renal failure or chronic diarrhea / dehydration.

## Discussion

This review shows that sodium lactate can be safely administrated to humans with insulin-induced hypoglycemia, TBI, Alzheimer’s disease, or heart failure. One study infused lactate in children, with the same minimal adverse effects as studies in adult populations. There are clear suggestions that the brain can use lactate as an energy source to compensate for the shortage of energy under conditions of hypoglycemia or as a potential therapeutic agent to limit the adverse consequences of TBI.

Nine studies that used lactate to induce panic attacks were included, although these studies did not use lactate infusion in a clinical setting. We decided to include these studies because of the possible risk to provoke panic attacks in future studies. However, these studies state that only patients with an anxiety disorder are susceptible to this provocation and a review of these studies reveals serious methodologic problems and the failure to consider cognitive variables. This renders the validity of these studies questionable [59]. Fortunately, panic attacks have not been reported in later studies involving healthy volunteers [60] and additionally, none of the other studies included in this review have reported panic attacks. It thus seems that the risk of panic attacks during lactate administration is small and, if at all present, restricted to patients with panic disorders.

Lactate infusion is not incorporated in standard care for any patient population. However, lactate infusion may be a possible treatment to improve the outcome of patients with TBI in the future [61–65]. To our knowledge, this review is the first to provide an overview of the therapeutic, albeit still experimental, use of lactate in clinical context. Previously, reviews outlining the basal metabolism of lactate have been published, these are beyond the scope of the current review [5, 7, 66–71]. Our review adds a focus on the clinical application of lactate infusion in humans by providing an overview of protocols used in literature to facilitate designing a protocol for future studies.

Additionally, it would be interesting to see if lactate could be used as an alternative energy source in children with a metabolic disorder, for example, Glut1DS, since the studies in this review suggest that lactate can be used by the adult brain as an alternative energy source. Difficulties in setting up a future study could be caused by the fact that so far only one study infused lactate in children. Little is known about the metabolism of lactate in children, and children are not little adults. So far, studies investigating the pharmacokinetic and pharmacodynamic properties of lactate infusion in pediatric patients have not been conducted. Obviously, such studies are of great importance to ensure safety of future clinical trials in children.

In conclusion, this review shows that lactate is an energy source for the adult brain. So far, one study investigated the infusion of lactate in children without major side effects. Additional studies to assess the safety of lactate infusion in children are needed, to allow future studies on its potential therapeutic effects, especially in children with rare inborn errors of metabolism. It will be also interesting to see if lactate infusion has beneficial effects in case of hypoglycemia (neuroglycopenia), TBI, or cardiac failure in pediatric populations. With this review, we hope to contribute to the discussion of lactate as potential therapeutic agent in children, and to facilitate future research.

## Supplementary information

Below is the link to the electronic supplementary material.

Supplementary file1 (DOCX 20 KB)

## Abbreviations

ANLS: Astrocyte neuron lactate shuttle
Glut1DS: Glucose transporter 1 deficiency syndrome
GSD1: Glycogen storage disease 1
TBI: Traumatic brain injury
