# Supplementary material for: Lactate infusion as therapeutical intervention: a scoping review
Source: Eur J Pediatr. 2022 Mar 18;181(6):2227–35. doi: 10.1007/s00431-022-04446-3 (PMC9110504; doi:10.1007/s00431-022-04446-3)
Supplement: Supplementary file 1 — Supplementary file1 (DOCX 20 KB) [file 431_2022_4446_MOESM1_ESM.docx]

PRISMA-ScR Checklist adapted from Tricco et al. (1)

| **No.** | **Item** | **Applied** | **Additional information** |
| --- | --- | --- | --- |
| **Title** | | | |
| 1 | Identify the report as a scoping review. | Yes |  |
| **Abstract** | | | |
| 2 | Provide a structured summary that includes (as applicable) background, objectives, eligibility criteria, sources of evidence, charting methods, results and conclusions that relate to the review questions and objectives. | Yes | Structured as provided by the journal. |
| **Introduction** | | | |
| 3 | Describe the rationale for the review in the context of what is already known. Explain why the review questions or objectives lend themselves to a scoping review approach. | Yes |  |
| 4 | Provide an explicit statement of the questions and objectives being addressed with reference to their key elements (for example, population or participants, concepts, and context) or other relevant key elements used to conceptualize the review questions or objectives. | Yes |  |
| **Methods** | | | |
| 5 | Indicate whether a review protocol exists; state if and where it can be accessed (for example, a Web address); and if available, provide registration information, including the registration number. | No | No protocol exists. |
| 6 | Specify characteristics of the sources of evidence used as eligibility criteria (for example, years considered, language, and publication status), and provide a rationale. | Yes |  |
| 7 | Describe all information sources in the search (for example, databases with dates of coverage and contact with authors to identify additional sources), as well as the date the most recent search was executed. | Yes |  |
| 8 | Present the full electronic search strategy for at least 1 database, including any limits used, such that it could be repeated. | Yes |  |
| 9 | State the process for selecting sources of evidence (that is, screening and eligibility) included in the scoping review. | No | Although not described in Methods, obviously all co-authors discussed the results as summarized in tables by first and last author. |
| 10 | Describe the methods of charting data from the included sources of evidence (for example, calibrated forms or forms that have been tested by the team before their use, and whether data charting was done independently or in duplicate) and any processes for obtaining and confirming data from investigators. | NA |  |
| 11 | List and define all variables for which data were sought and any assumptions and simplifications made. | Yes |  |
| 12 | If done, provide a rationale for conducting a critical appraisal of included sources of evidence; describe the methods used and how this information was used in any data synthesis (if appropriate). | NA |  |
| 13 | Summary measures, not applicable for scoping reviews. | NA |  |
| 14 | Describe the methods of handling and summarizing the data that were charted. | NA |  |
| 15 | Risk of bias across studies, not applicable for scoping reviews. | NA |  |
| 16 | Additional analyses, not applicable for scoping reviews. | NA |  |
| **Results** | | | |
| 17 | Give numbers of sources of evidence screened, assessed for eligibility, and included in the review, with reasons for exclusions at each stage, ideally using a flow diagram. | Yes |  |
| 18 | For each source of evidence, present characteristics for which data were charted and provide the citations. | NA |  |
| 19 | If done, present data on critical appraisal of included sources of evidence (see item 12). | NA |  |
| 20 | For each included source of evidence, present the relevant data that were charted that relate to the review questions and objectives. | Yes |  |
| 21 | Summarize or present the charting results as they relate to the review questions and objectives. | Yes |  |
| 22 | Risk of bias across studies, not applicable for scoping reviews. | NA |  |
| 23 | Additional analyses, not applicable for scoping reviews. | NA |  |
| **Discussion** | | | |
| 24 | Summarize the main results (including an overview of concepts, themes, and types of evidence available), link to the review questions and objectives, and consider the relevance to key groups. | Yes |  |
| 25 | Discuss the limitations of the scoping review process. | Yes |  |
| 26 | Provide a general interpretation of the results with respect to the review questions and objectives, as well as potential implications or next steps. | Yes |  |
| **Funding** | | | |
| 27 | Describe sources of funding for the included sources of evidence, as well as sources of funding for the scoping review. Describe the role of the funders of the scoping review. | Yes |  |

Search

("lactic acid"[MeSH Terms] OR ("lactic"[tiab] AND "acid"[tiab]) OR "lactic acid"[tiab] OR "lactate"[tiab] OR "lactates"[MeSH Terms] OR "lactates"[tiab]) AND infusion[All Fields] AND (("brain"[MeSH Terms] OR "brain"[All Fields]) OR ("cerebrum"[MeSH Terms] OR "cerebrum"[All Fields])) AND ("humans"[MeSH Terms] AND (Dutch[lang] OR English[lang]))

References

1. Tricco AC, Lillie E, Zarin W, O'Brien KK, Colquhoun H, Levac D, et al. PRISMA Extension for Scoping Reviews (PRISMA-ScR): Checklist and Explanation. Ann Intern Med. 2018;169(7):467-73.
